# Reconstruction of cervical and upper thoracic esophagus with a free posterior tibial artery perforator flap: A case report

**DOI:** 10.1097/MD.0000000000022617

**Published:** 2020-11-13

**Authors:** Jun Liu, Jifeng Liu, Jianjun Ren, Ji Wang, Dan Lv, Di Deng, Linke Li, Fei Chen

**Affiliations:** Department of Otorhinolaryngology, Head and Neck Surgery, West China Hospital, Sichuan University, Chengdu, China.

**Keywords:** case report, esophagoplasty, free posterior tibial artery perforator flap

## Abstract

**Introduction:**

Esophageal window defect in patients with esophageal resection could be challenging to repair. In this case report, a free posterior tibial artery perforator flap (FPTAPF) was used for semi-circumference patch esophagoplasty.

**Patient concerns:**

For this 47-year-old male patient with recurrent laryngeal nerve schwannoma invading cervical and upper thoracic esophagus, cervical and upper thoracic esophageal reconstruction following tumor resection was needed

**Diagnosis:**

Pathologic result demonstrated recurrent laryngeal nerve schwannoma. Ultrasound examination detected a tumor (7 cm × 6 cm × 3 cm) located behind the right thyroid lobe, and contrast-enhanced computed tomography scan revealed that tumor was located between the cervical esophagus and trachea, and compressed these structures.

**Interventions:**

The tumor had a size of 7 cm × 6 cm × 3 cm, and the semi-circumference defect of the cervical and upper thoracic esophagus was about 7 cm in length after complete tumor resection. A 7 cm × 4 cm FPTAPF was designed and harvested for esophageal reconstruction.

**Outcomes:**

The posterior tibial flap survived well and satisfactory recovery of esophageal function was obtained with no significant complications. No local tumor relapse was indicated by computed tomography during the 2-year postoperative follow-up.

**Conclusion:**

This case highlights the stable performance of FPTAPF when used for the reconstruction of large esophageal window defect.

## 1 Introduction

Schwannoma rarely originates from recurrent laryngeal nerve in clinical practice.^[1]^ For patients with recurrent laryngeal nerve schwannoma, surgical resection of tumor, which could help to relieve esophageal obstruction, is critical to the initial management, especially when the tumor invaded cervical and upper thoracic esophageal mucosa. However, esophageal defect after esophageal window resection could be too large to be reconstructed directly by traditional methods (eg, the pedicled flap and free flap).^[2,3]^ Until now, this constructive procedure has been a great challenge for head and neck surgeons. Owing to bulky volume, the pedicled flap was difficult to reconstruct the esophageal defect.^[4]^ The tubular autografts (eg, stomach, colon, and jejunum) were widely used for esophageal reconstruction. Nevertheless, these autografts utilized to reconstruct the circumferential esophageal defect might not be suitable for larger esophageal window defect. Additionally, the harvest of autograft is time consuming and more technical demanding.^[5,6]^ Compared with free radial forearm flap, the free posterior tibial artery perforator flap (FPTAPF) with similar tissue properties and hidden scar has been used to reconstruct the head and neck defect. Here we report a case in which the FPTAPF was used for the reconstruction of large esophageal window defect caused by resection of recurrent laryngeal nerve schwannoma invading esophagus.

## 2 Case report

**Figure 1:**
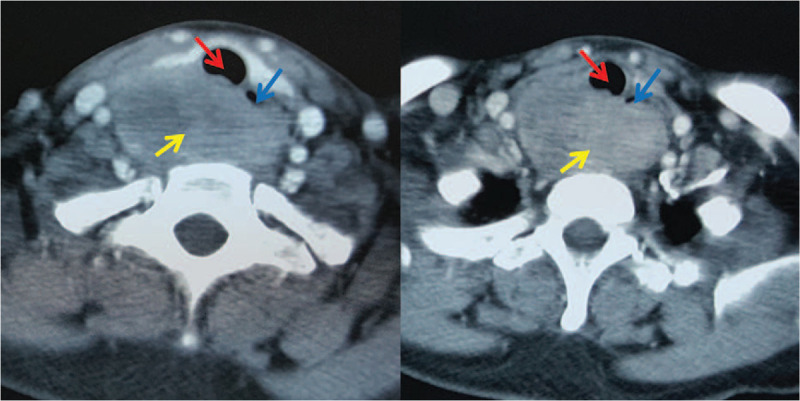
Computed tomography scan: the tumor was located between the cervical esophagus and trachea, and compressed them. Note: tumor (yellow arrow); trachea (red arrow); esophagus (blue arrow).

A 47-year-old male patient with neck mass for 3 years, progressive dysphagia lasting over 6 months and difficulty breathing after exercise for more than 1 month was referred to our department. Ultrasound examination detected a tumor (7 cm × 6 cm × 3 cm) located behind the right thyroid lobe. Preoperative biopsy indicated a spindle cell tumor. Contrast-enhanced computed tomography scan revealed that the tumor was located between the cervical esophagus and trachea, and compressed these structures (Fig. 1).

**Figure 2:**
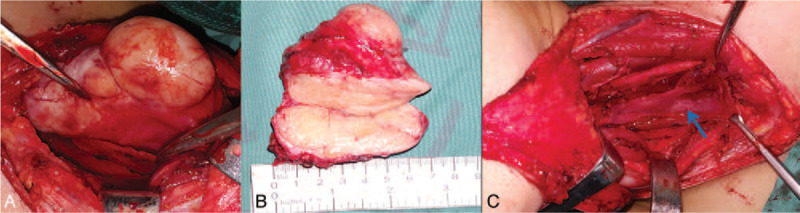
Tumor resection. A: The tumor (7 cm × 6cm × 3 cm) was exposed, which originated from the right recurrent laryngeal nerve and invaded the esophageal mucosa; B and C: The half circumference defect of the esophagus was about 7 cm in length after complete tumor resection. Note: residual esophagus (blue arrow).

Tracheal intubation and general anesthesia were performed. A U-shaped skin flap was elevated to the level of hyoid bone and the right carotid sheath was exposed and pulled outward. The tumor (7 cm × 6 cm × 3 cm) originated from the right recurrent laryngeal nerve and invaded the esophageal mucosa (Fig. 2A, B). The tumor, partial right recurrent laryngeal nerve, partial cervical and upper thoracic esophagus were resected. Rapid intraoperative pathological examination indicated a spindle cell tumor. Meanwhile, the tumor-free margins were confirmed by intraoperative frozen section pathology analysis. The semi-circumference defect of the esophagus was about 7 cm in length after complete tumor resection (Fig. 2C).

**Figure 3:**
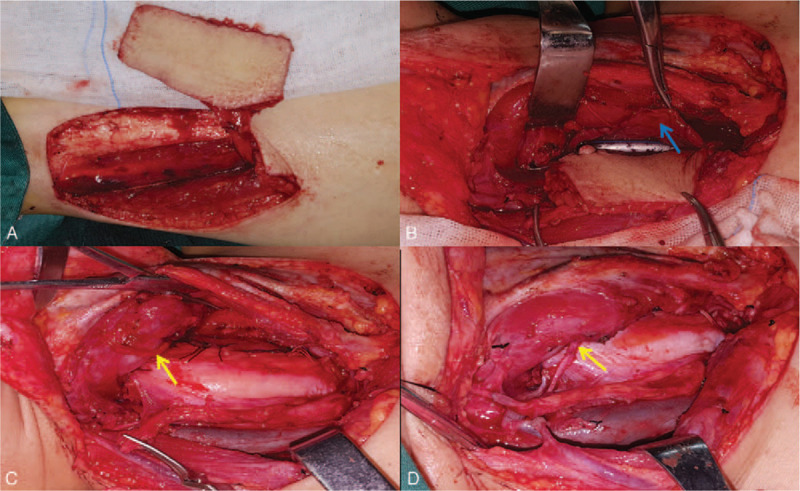
A: Harvesting of a free posterior tibial artery perforator flap; B and C: Reconstruction of esophageal defect using a free posterior tibial artery perforator flap; D: Microvascular anastomosis and right recurrent laryngeal nerve reconstruction using the right ansa cervicalis. Note: residual esophagus (blue arrow), the residual right recurrent laryngeal nerve entering larynx (yellow arrow).

Passing through the hypopharynx and down into the distal esophagus, a nasogastric tube was inserted. A FPTAPF with the size of 7 cm × 4 cm, was designed and harvested to reconstruct the esophageal window defect (Fig. 3A). Once the cervical recipient vessels (including superior thyroid artery, superior thyroid vein, and external jugular vein) were prepared, the FPTAPF was transferred to the defect site. As shown in Fig. 3B, C, and D, the flap was initially sutured to the residual esophageal mucosa. Microvascular anastomosis, during which 1 artery and 2 veins were anastomosed, was then performed. In addition, the part of right recurrent laryngeal nerve entering larynx was identified. The right ansa cervicalis was used to restore the continuity of the right recurrent laryngeal nerve. A tracheotomy was carried out before skin closure.

**Figure 4:**
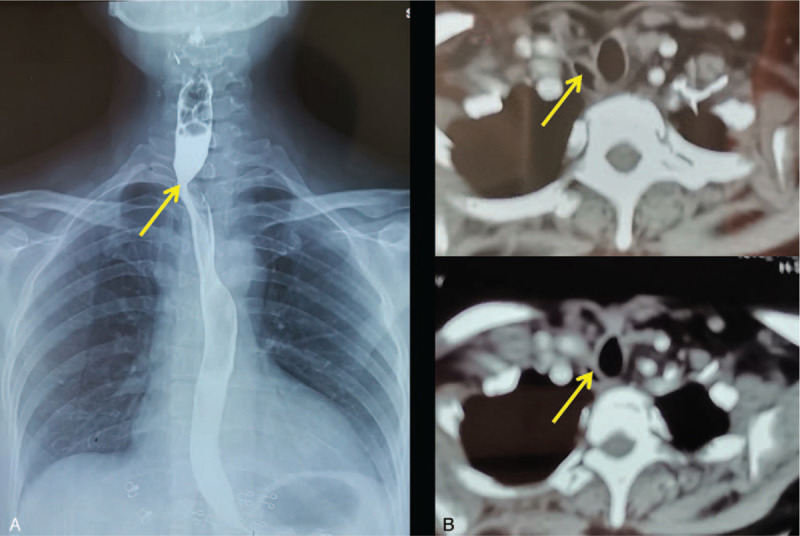
Postoperative esophagography and CT scan. A: Esophagography shows the function of reconstructed esophagus at the third month postoperatively; B: CT shows the reconstructed esophagus at the second year postoperatively. Note: reconstructed esophagus (yellow arrow).

The operative time of the procedure was about 3 hours. The flap survived well postoperatively with no operation-related complication observed. Postoperative pathological examination results showed that the tumor was a recurrent laryngeal nerve schwannoma, and invaded the esophageal mucosa. Oral feeding and normal diet were resumed on postoperative day 22 and day 35, respectively. Satisfactory recovery of swallowing function was gained. The patient was weaned from tracheostomy on postoperative day 60. An excellent function of the reconstructed esophagus was observed by esophagography on postoperative day 90 (Fig. 4A) and no local tumor relapse was indicated by computed tomography during the 2-year postoperative follow-up (Fig. 4B). Voice hoarseness existed postoperatively but relieved significantly within 3 months after operation.

The report was approved by the Institutional Review Board of West China Hospital. Written informed consent was obtained from the patient for publication of this case report and accompanying images.

## 3 Discussion

As a benign neurogenic tumor, schwannnomas arise from schwann cells surrounding peripheral nerves and up to 45% of schwannomas occur in head and neck region,^[1]^ which mainly arise from the sympathetic nerve. In the present case, schwannoma arising from the right recurrent laryngeal nerve, is quite rare. Therapeutic management is mainly based on surgery. However, when giant recurrent laryngeal nerve schwannnomas invaded the esophagus, the reconstruction of large esophageal window defect after extensive tumor resection would be a great challenge. The esophagus should be reconstructed to restore its integrity after partial resection.

In recent decades, the pedicled and free flaps (eg, pectoralis major myocutaneous, supraclavicular, free radial forearm and adipofascial anterolateral thigh free flap) were widely used to reconstruct hypopharyngeal and esophageal defects.^[7,8]^ However, such thick free or pedicle flaps might not be feasible for repairing defects in the narrow space around esophageal defects under the preservation of larynx and trachea. Therefore, thinner free flaps remain a better choice for the reconstruction of large esophageal window defects. Free radial forearm flap and adipofascial anterolateral thigh free flap have been used to reconstruct esophageal defects, but not semi-circumference ones, and satisfactory reconstruction of esophageal function was obtained.^[7,8]^ It has been reported that free posterior tibial artery perforator flaps have several unique advantages. The constant vascular anatomy on the medial surface of shank of septocutaneous perforators of posterior tibial artery makes its access much easier than other fasciocutaneous flaps.^[9]^ More importantly, a FPTAPF can also provide similar tissue properties to a free radial forearm flap, which has been widely and successfully used in reconstruction. In addition, compared to free radial forearm flap, FPTAPF with hidden scar of flap donor region has incomparable advantage for aesthetical consideration.^[10,11]^ Meanwhile, FPTAPFs have the potential of reconstruction of larger defect than radial forearm flap. We have reported in a case report that, FPTAPF, specifically a free bipaddled posterior tibial artery perforator flap with size of 12 cm × 9 cm was successfully used for simultaneous tracheal and esophageal reconstruction for thyroid cancer involving trachea and esophagus.^[12]^ However, patients with severe peripheral artery disease or varicose veins of the lower limb were not suggested to undergo FPTAPF surgery.

In the current case, we described a novel use of FPTAPF for semi-circumference patch esophagoplasty in a patient with recurrent laryngeal nerve schwannoma invading cervical and upper thoracic esophagus, which was under different situation with previously reported case in terms of tumor and invasion status. As a result, the posterior tibial flap survived well, and esophageal function was recovered with no significant complications. We believe that this case might stable performance of FPTAPF when used for the reconstruction of large esophageal window defect.

## Author contributions

**Conceptualization:** Jun Liu, Jifeng Liu, Jianjun Ren, Linke Li, Fei Chen.

**Data curation:** Jifeng Liu, Jianjun Ren, Ji Wang, Dan Lv, Di Deng, Linke Li, Fei Chen.

**Formal analysis:** Ji Wang, Dan Lv.

**Funding acquisition:** Jun Liu, Fei Chen.

**Investigation:** Jun Liu, Ji Wang, Di Deng, Linke Li, Fei Chen.

**Methodology:** Jun Liu, Jifeng Liu, Jianjun Ren, Ji Wang, Dan Lv, Di Deng, Linke Li, Fei Chen.

**Resources:** Di Deng.

**Supervision:** Jifeng Liu, Jianjun Ren, Fei Chen.

**Validation:** Dan Lv.

**Writing – original draft:** Jun Liu, Jifeng Liu, Jianjun Ren, Dan Lv, Fei Chen.

**Writing – review & editing:** Jun Liu, Jifeng Liu, Jianjun Ren, Di Deng, Fei Chen.
